# Multimodal neuroimaging of *Col4a1*-mutant mouse models of Gould syndrome

**DOI:** 10.3389/fnins.2025.1639871

**Published:** 2025-11-12

**Authors:** Xiao Gao, Xiaowei Wang, Cassandre Labelle-Dumais, Douglas B. Gould, Myriam M. Chaumeil

**Affiliations:** 1 Department of Physical Therapy and Rehabilitation Science, UCSF, San Francisco, CA, United States; 2 Department of Radiology and Biomedical Imaging, UCSF, San Francisco, CA, United States; 3 UC Berkeley-UCSF Graduate Program in Bioengineering, UCSF, San Francisco, CA, United States; 4 Department of Ophthalmology, UCSF, San Francisco, CA, United States; 5 Department of Anatomy, Institute for Human Genetics Bakar Aging Research Institute, Cardiovascular Research Institute, UCSF, San Francisco, CA, United States

**Keywords:** MRI, Gould syndrome, collagen, *Col4a1*, vascular dementia, cSVD

## Abstract

**Introduction:**

Cerebral small vessel disease (cSVD) is a leading cause of stroke and vascular contributions to cognitive impairment and dementia (VCID). Studying monogenic forms of cSVD can elucidate molecular pathways that are dysfunctional in the common sporadic forms and may serve as potential therapeutic targets. Mutations in *COL4A1* and *COL4A2* cause highly penetrant cSVD as part of the multisystem disorder known as Gould syndrome, which includes cerebrovascular manifestations such as porencephaly, early-onset stroke, leukoencephalopathy, and intracerebral hemorrhage (ICH).

**Methods:**

To investigate how allelic heterogeneity influences cerebrovascular phenotypes, we examined five *Col4a1* mutant mouse strains that collectively model the clinical spectrum of Gould syndrome. Each strain underwent multimodal magnetic resonance imaging (MRI) at 14.1 Tesla to assess radiological features characteristic of cSVD.

**Results:**

Multimodal MRI successfully identified typical cSVD-associated lesions across all *Col4a1* mutant strains. The imaging revealed heterogeneous expressivity among the allelic variants in terms of lesion prevalence, size, and number. Furthermore, analysis across strains identified brain regions that were consistently more vulnerable to cSVD-related lesions.

**Discussion:**

These findings demonstrate that high-field multimodal MRI can sensitively detect and differentiate cerebrovascular abnormalities among *Col4a1* mutant mouse models of Gould syndrome. The approach provides a powerful, noninvasive platform for assessing genotype–phenotype relationships and for identifying brain regions at heightened risk in cSVD, supporting its potential use in early diagnosis and mechanistic studies of vascular pathology.

## Introduction

Cerebral small vessel disease (cSVD) is a group of cerebrovascular conditions that account for up to 30% of strokes and are a major cause of vascular contributions to cognitive impairment and dementia (VCID)—an irreversible and progressive form of dementia that has become a significant public health issue (Petty et al., 2000; Bamford et al., 1987; Pantoni, 2010; Pantoni and Gorelick, 2014). Monogenic and idiopathic forms of cSVDs (Rutten-Jacobs et al., 2015; Yamamoto et al., 2011; Choi, 2015; Joutel et al., 2016; Joutel and Faraci, 2014) share clinical manifestations, suggesting common pathogenic mechanisms. Monogenic forms of complex disorders tend to be more severe and occur at younger ages. Thus, monogenic forms of cSVD may represent experimentally tractable settings to provide insight into pathogenic processes underlying common age-related disease subtypes. The genes encoding type IV collagen alpha 1 (*COL4A1*) and alpha 2 (*COL4A2*) share a common genetic locus that is reproducibly associated with cSVD hallmarks in large-scale genetic studies including white matter hyperintensities (Malik et al., 2018; Persyn, 2020; Sargurupremraj et al., 2020; Traylor et al., 2021; Mishra et al., 2022; Duperron et al., 2023), enlarged perivascular spaces (Duperron et al., 2023), ischemic stroke (Mishra et al., 2022), and small vessel stroke (Mishra et al., 2022), even after accounting for hypertension (Sargurupremraj et al., 2020), making *COL4A1/A2* mutations a promising study subject for revealing the disease-causing mechanisms that eventually lead to cSVD.

COL4A1 and COL4A2 assemble into heterotrimers [α1α1α2(IV)] that are conserved throughout the animal kingdom and are fundamental constituents of specialized extracellular matrix structures called basement membranes. Rare, dominant, coding mutations of *COL4A1* and *COL4A2* cause monogenic cerebrovascular disease as part of a highly variable multisystem disorder named Gould syndrome. CSVD is one of the most notable features of Gould syndrome and encompasses a constellation of clinical manifestations, including porencephaly, early-onset stroke, leukoencephalopathy, intracranial aneurysms, and recurrent intracerebral hemorrhage (ICH) (Breedveld et al., 2006; Gould et al., 2005; Gould et al., 2006; Sibon et al., 2007; Guey and Hervé, 2022). Missense mutations of highly conserved glycine residues in the COL4A1 and COL4A2 triple helical domain represent the most prevalent class of mutations (Jeanne et al., 2015; Whittaker et al., 2022). Murine models of *Col4a1* and *Col4a2* mutations faithfully replicate Gould syndrome and allelic heterogeneity was shown to modulate the severity of ICH in an allelic series of *Col4a1* and *Col4a2* mutant mice (Jeanne and Gould, 2017; Labelle-Dumais, 2019; Kuo et al., 2014).

While the qualitative diagnosis of Gould syndrome relies on molecular genetic analysis (Sondergaard et al., 2017; Guey et al., 2021), neuroradiology, in particular magnetic resonance imaging (MRI), provides valuable early detection and monitorization of the progression of cSVD, serving as an indispensable tool for timely clinical intervention. The typical neuroimaging findings of cSVD include white matter lesions, lacunar infarcts, and hemorrhagic lesions, which can be readily detected by different MRI techniques, such as T2-weighted imaging (T2WI), Fluid Attenuated Inversion Recovery (FLAIR), and susceptibility-weighted imaging (SWI). Allelic heterogeneity in *Col4a1* mutant mice offers an opportunity to develop and evaluate multimodal imaging for studying the breadth of phenotypes in this model of monogenic cSVD. This may provide insight into correlations between genotypes, radiological features, and histopathology findings, which can improve the understanding of cSVD generally and Gould syndrome more specifically.

**Figure 1:**
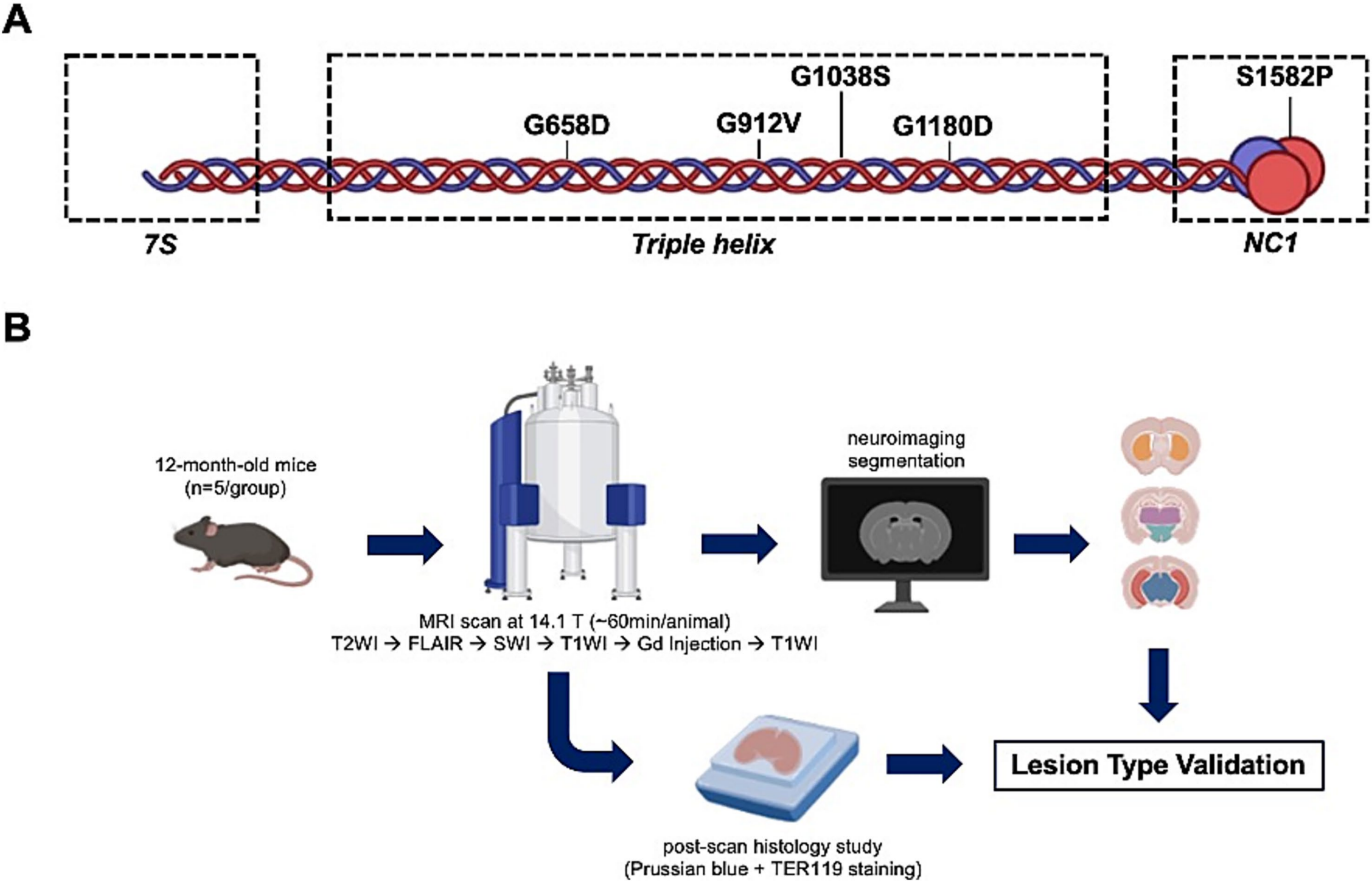
Overall study design. **(A)** Schematic of the structure of the collagen IV molecule (triple helix) and five *Col4a1* missense mutations studied. Type IV collagens contain three major structural domains: the 7S, the triple helix (collagenous) and the globular non-collagenous 1 (NC1). **(B)** Schematic of the multimodal MRI study workflow. FLAIR, Fluid-Attenuated Inversion Recovery Imaging; Gd, Gadolinium; SWI, Susceptibility-Weighted Imaging; T2WI, T2-Weighted Imaging; T1WI, T1-Weighted Imaging. Created with BioRender.com.

In this study, we used five different *Col4a1* mutant mouse strains that recapitulate the clinical spectrum of cerebrovascular manifestations associated with Gould syndrome (Figure 1A). Using this unique allelic series, we characterized the neuroradiology features of cerebrovascular lesions using multimodal 14.1 Tesla MRI. We established a machine learning-based imaging analysis pipeline to quantify radiological changes and used *ex vivo* studies to elucidate the pathological basis of the respective MR lesions (Figure 1B). Overall, our study demonstrates that multimodal MRI combined with machine learning successfully identify typical cSVD lesions and heterogeneous expressivity in a *Col4a1* murine allelic series in terms of anatomical changes, and lesion prevalence, number and volume.

## Results

The primary goal of this study was to optimize multimodal MRI pipeline to detect lesions and disease burden in mouse models of a monogenic form of cSVD. To maximize the potential to detect and characterize cSVD lesions, we chose to investigate old animals (52.1 ± 1.26 weeks) as the severity of cerebrovascular manifestations is exacerbated with age in *Col4a1* mutant mice (Jeanne et al., 2015).

We used 5 *Col4a1* mutant strains that well mimic the disease spectrum observed in individuals with Gould syndrome. Each strain carries a distinct mutation, including 4 different glycine missense mutations in the triple helical domain (*Col4a1^+/G658D^*, *Col4a1^+/G912V^*, *Col4a1^+/G1038S^*, and *Col4a1^+/G1180D^*) and one mutation in the NC1 domain (*Col4a1^+/S1582P^*). While there was no difference in age across all animal groups (Supplementary Figure S1A), 3 out 5 strains have reduced body weight compared to WT (*Col4a1^+/+^*; Supplementary Figure S1B), which is consistent with previous report of reduced body size in Col4a1 mutant mice and might contribute to potential change in overall brain size.

### T2WI and FLAIR MRI reveals variable volumetric changes in *Col4a1* mutant brains

**Figure 2:**
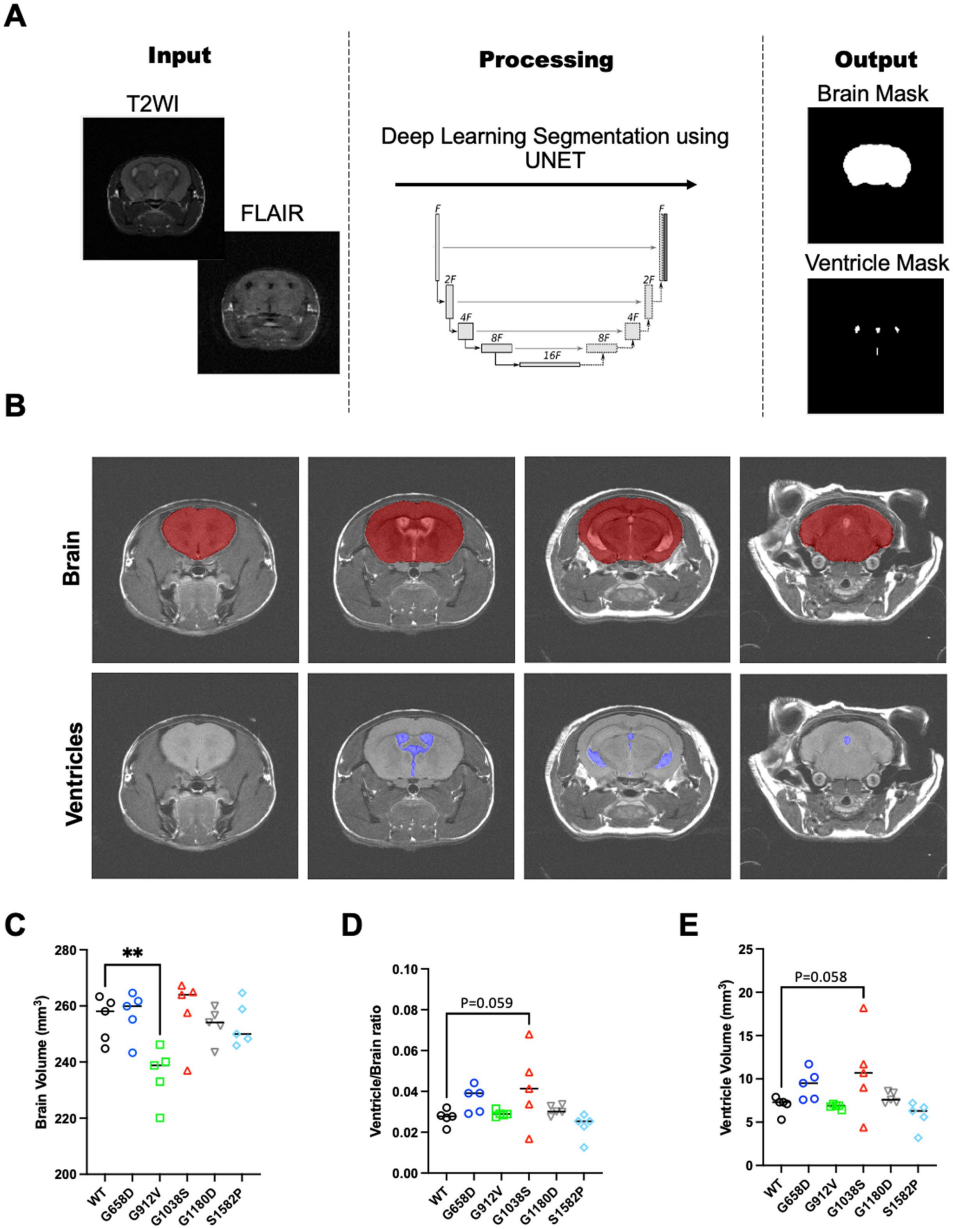
Volumetric analysis using U-net. **(A)** Schematic of the convolutional neural networks (U-net) implemented: (1) skull-stripping U-net for extraction of whole brain volume; (2) tissue/lesion-specific U-net. **(B)** Representative segmentation of skull-stripping and ventricle-segmenting U-net. Quantification of **(C)** Brain volume, **(D)** ventricle volume, and **(E)** ventricle-to-brain ratios in mouse models of Gould syndrome. Brain volume was significantly lower in *Col4a1^+/G912V^* mice (G912V) compared to *Col4a1^+/+^* (WT) controls (**p < 0.01). *Col4a1^+/G1038S^* (G1038S) showed a tendency for increased ventricle volume and ventricle-to-brain ratio compared to WT controls (*p* = 0.059 and 0.058, respectively).

The pipeline for U-net segmentation of brain and ventricle volumes is shown in Figure 2A. Typical results of both parenchyma and ventricle segmentations are shown in Figure 2B. The quality control (QC) passing rate of skull-stripping output ranged from 82 to 94% depending on the input MRI sequences, while the Dice Coefficient maintained above 0.94 for both QC-passed and -failed cases (Supplementary Table S1). The brain slices with QC-failed skull-stripping either contained extremely large brain lesions or were located at the rostral-caudal ends (Supplementary Figure S2). Upon quantification, our results show that whole brain volume was significantly lower in *Col4a1^+/G912V^* mice than in WT controls (**p = 0.009, Figure 2C). In contrast, *Col4a1^+/G1038S^* mice exhibited a trend toward larger ventricle volume as well as ventricle-to-brain ratios compared to WT controls (*p* = 0.059 and 0.058; Figures 2D,E). No other significant differences in ventricle or brain volumes were observed for the rest genotypes.

**Figure 3:**
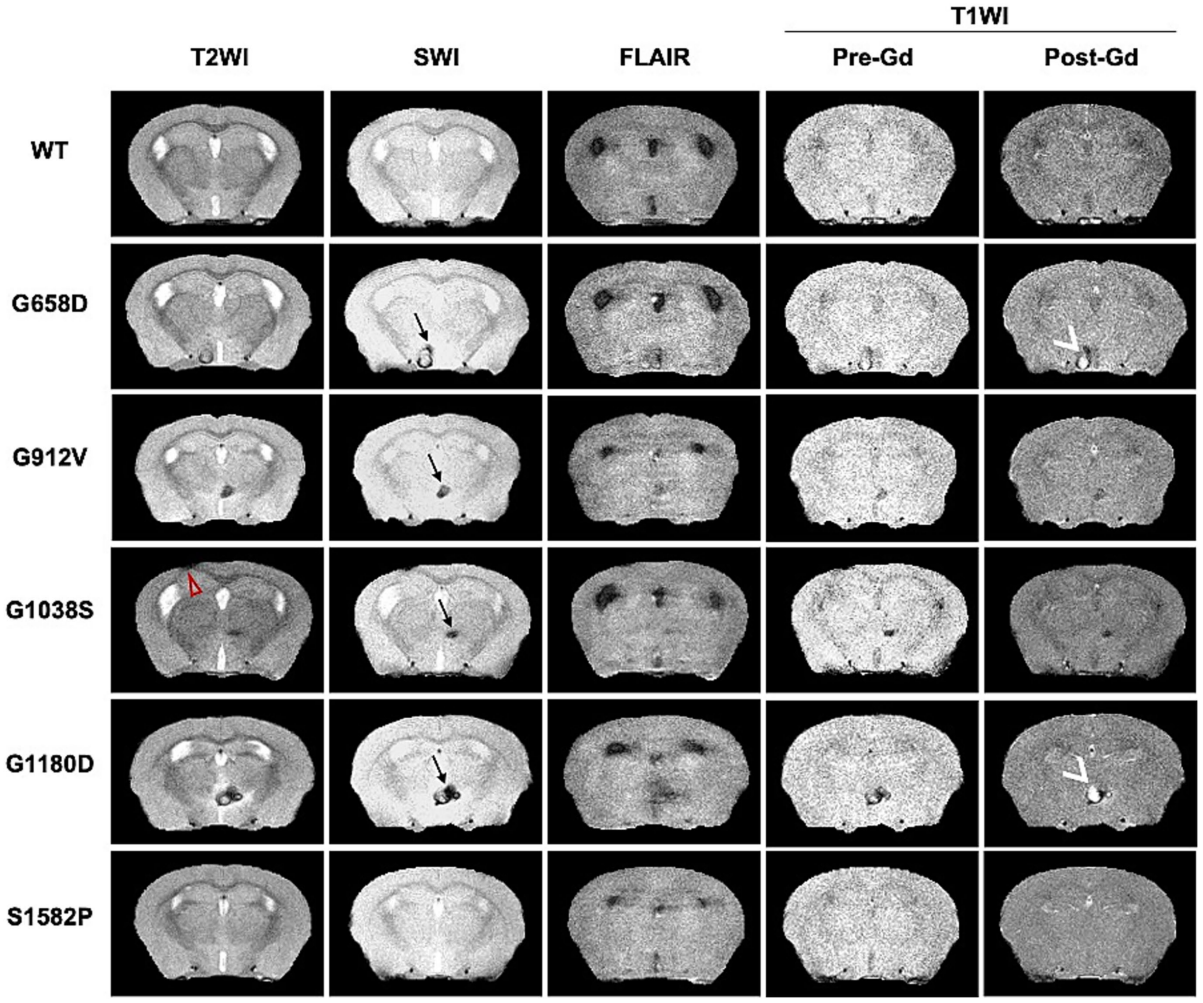
Representative multimodal MRI of *Col4a1* mutant and wild-type mice acquired at 14.1 Tesla. Representative images of all five different MRI modalities are shown for all five genotypes (after skull-striping via U-net). SWI-positive lesions (black →) were detected in all *Col4a1* mutant strains, but not in WT mice. Increased post-Gd enhancement (white >) was only found in *Col4a1^+/G658D^* (G658D), *Col4a1^+/G1038S^* (G1038S), and *Col4a1^+/G1180D^* (G1180D) by T1WI. One schizencephaly case was found in *Col4a1^+/G1038S^* mice and indicated by red ▻ in T2WI.

Representative brain images after skull-stripping are also shown for all five genotypes and all five MRI modalities used in this study (Figure 3) to demonstrate the phenotypic variability of radiological manifestations among *Col4a1* mutant mice. Of note, one mouse from one strain (*Col4a1^+/G1038S^*) presented SWI sensitive lesions along with schizencephaly (red arrow in Figure 3), a radiological features associated with Gould syndrome (Yoneda, 2013; Matsumoto, 2015; Smigiel, 2016; Khalid, 2018). In three out of five genotypes (*Col4a1^+/G658D^*, *Col4a1^+/G1180D^*, and *Col4a1^+/G1038S^*), T1WI showed gadolinium (Gd) enhanced lesions. These results indicate that *Col4a1* mutations can lead to both macro- and micro-scopic structural changes detectable by MRI.

### Hypointense lesions detected by SWI vary in prevalence, number, volume, and type in the allelic series of *Col4a1* mutant mice

The summary of prevalence, number, volume, and type of cerebral lesions observed in *Col4a1* mutant mice is shown in Table 1. There was a range in the prevalence of SWI-positive lesions among *Col4a1* mutant mice, from 20% for *Col4a1^+/S1582P^* mice to 100% for *Col4a1^+/G1038S^* mice. When exclusively considering animals with SWI-positive lesions, the individual lesion burden also shared noteworthy variability, where *Col4a1^+/G1038S^* mice had 15.8 ± 6 lesions per animal while only two lesions were found in a single *Col4a1^+/S1582P^* mouse. These data are consistent with mutations in the NC1 domain leading to milder pathology (Kuo et al., 2014). We also observed that *Col4a1^+/G1038S^* mice presented with large isolated lesions (16.3 ± 9 × 10^−1^ mm^3^ per lesion), which is consistent with age-related macrohemorrhages reported previously (Ratelade et al., 2018; Branyan et al., 2023).

**Table 1:** Prevalence, number, volume and type of SWI-positive lesions in *Col4a1* mutant mouse strains.

Genotype	Prevalence of lesions	Number of lesions per positive cases (mean ± s.d.)	Volume of lesions per positive case (mm^3^, mean ± s.d.)	% of lesions with Gd enhancement
WT	0 of 5 (0%)	0.0 ± N/A	0.0 ± N/A	N/A
G658D	3 of 5 (60%)	1.3 ± 0.6	1.6 ± 2.8 × 10^−1^	25%
G912V	3 of 5 (60%)	3.0 ± 1.0	1.1 ± 1.1 × 10^−1^	0%
G1038S	5 of 5 (100%)	15.8 ± 6.0	16.3 ± 9.0 × 10^−1^	1.3%
G1180D	3 of 5 (60%)	3.3 ± 2.5	3.9 ± 5.2 × 10^−1^	20%
S1582P	1 of 5 (20%)	2 ± N/A	1.7 ± 3.7 × 10^−1^	0%

Additionally, we investigated the percentage of SWI-positive lesions that presented Gd enhancement, i.e., Gd(+), as detected by T1WI. Interestingly, these percentages were once again highly variable between genotypes, ranging from no enhancing lesions in *Col4a1^+/S1582P^* and *Col4a1^+/G912V^* mice, to 20 and 25% in *Col4a1^+/G1180D^* and *Col4a1^+/G658D^* mice, respectively. Notably, in *Col4a1^+/G1038S^* mice, the genotype presenting the highest lesion burden and largest solitary lesion size, only 1.3% of lesions were Gd(+).

### Regional analysis of SWI-positive lesions emphasizes the vulnerability of deep grey matter areas

**Figure 4:**
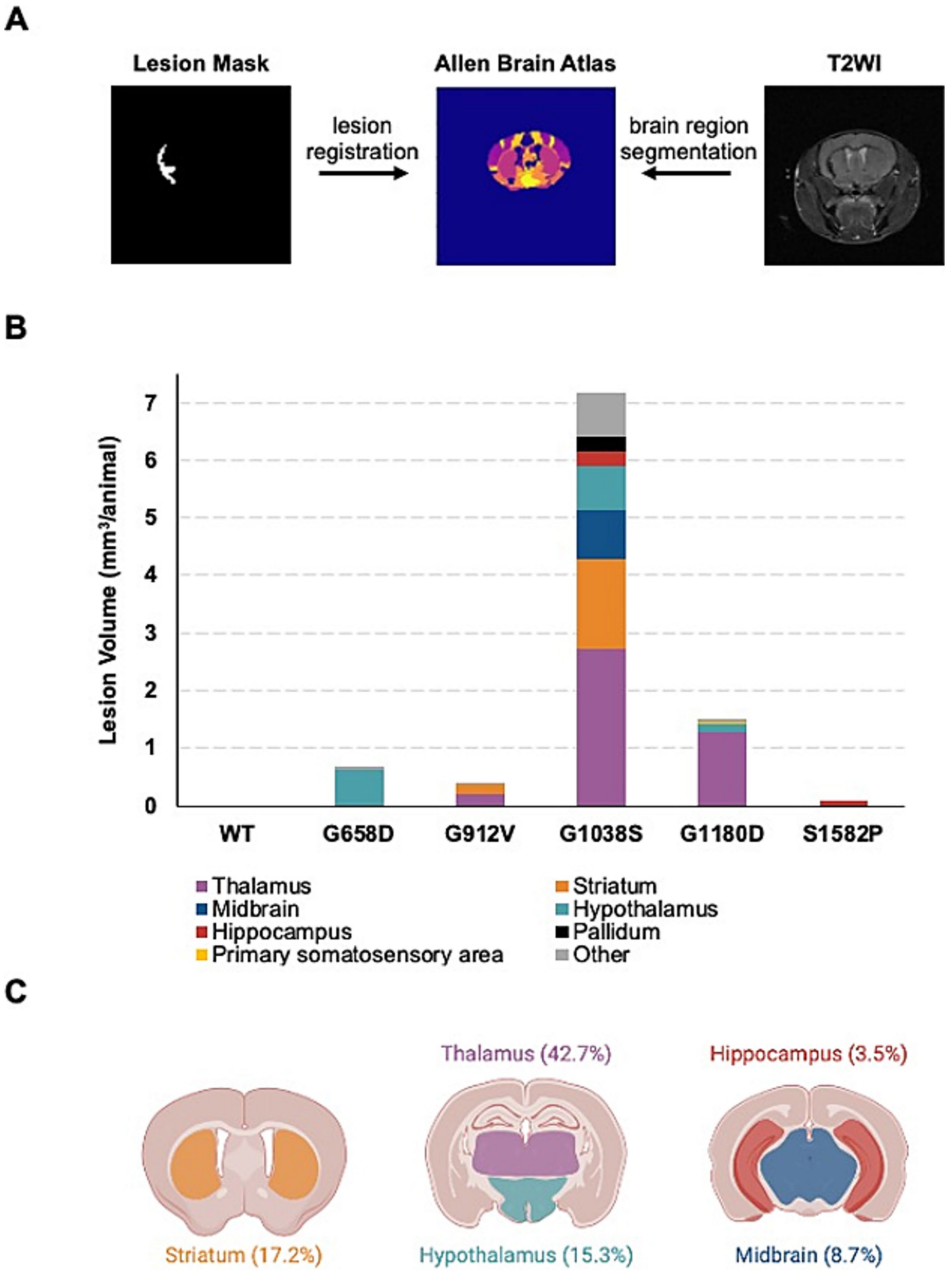
Regional analysis of SWI-positive lesions. **(A)** Schematic of co-registration of brain regions and SWI-positive lesions on the Allen Brain Atlas. **(B)** Sum of lesion volume per genotype and regional distribution of lesions based on co-registration with Allen Brain Atlas. **(C)** Distribution of all lesions in *Col4a1* mutant mice, irrespective of genotype. More than 85% of brain lesions can be found in the thalamus (42.7%), striatum (17.2%), hypothalamus (15.3%), midbrain (8.7%), and/or hippocampus (3.5%). Mouse brain illustration created with BioRender.com.

We co-registered the hypointense lesions from SWI and the anatomical structures from T2WI according to the defined anatomical structure in Allen Mouse Brain Atlas (Figure 4A). Analysis of SWI-positive lesions showed deep grey matter areas were compromised in all five *Col4a1* mutant strains, with the top affected regions from the most to least frequent being the thalamus (42.7%), striatum (17.2%), hypothalamus (15.3%), midbrain (8.7%), and hippocampus (3.5%; Figure 4C). *Col4a1^+/G1038S^* mice, showed larger lesions, and had a broader range of brain regions affected, including lesions in the midbrain (Figure 4B).

Combining SWI and T1-post Gd MRI enables differentiation between distinct types of lesions.

**Figure 5:**
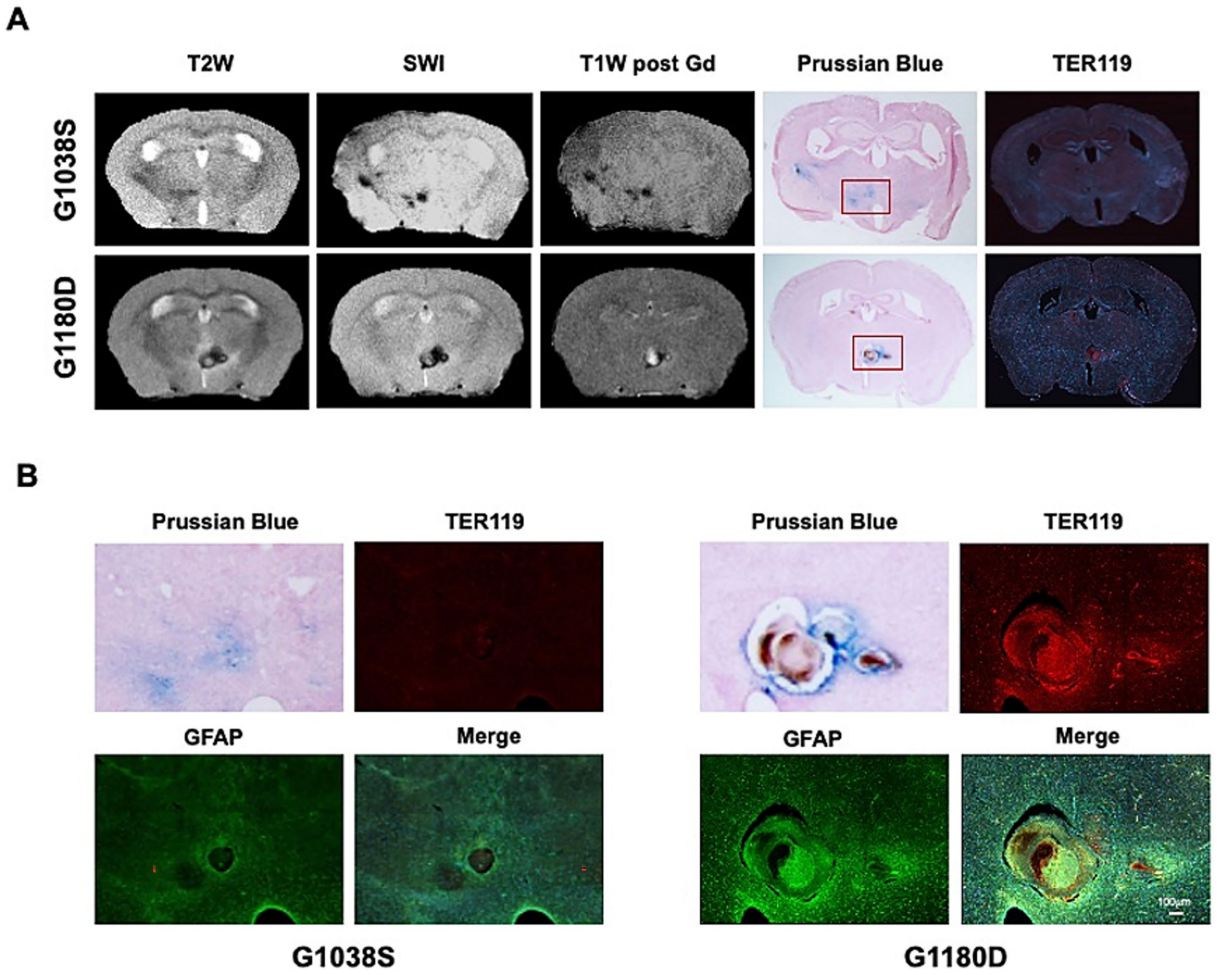
Characterization of Gd(−) vs. Gd(+) SWI-positive lesions in *Col4a1* mutant mice. **(A)** MRI, Prussian Blue staining, and TER119 immunolabeling images of Gd(−) vs. Gd(+) SWI-positive lesions, represented by a *Col4a1^+/G1038S^* (G1038S) case (top row) and a *Col4a1^+/G1180D^* (G1180D) case (bottom row). **(B)** Zoom-in outlook of lesions, showing Prussian blue staining, and co-immunolabeling for TER119 and GFAP.

As noted, not all SWI-positive lesions were Gd(+), as detected by enhanced T1WI, and the percentage was variable between genotypes. To better understand the difference of these lesions, we performed Perl’s Prussian blue staining and immunolabeling for TER-119 to test for the presence of iron deposition and red blood cells, respectively in one brain with Gd(+) SWI-positive lesion (*Col4a1^+/G1180D^*) and one brain with Gd(−) SWI-positive lesion (*Col4a1^+/G1038S^*). In both lesions, iron deposition was detected by Perl’s Prussian Blue staining, as shown in Figure 5A. However, only the Gd(+) lesion was filled with non-degraded red blood cells as indicated by the high TER-119 immunopositive signal, suggestive of a hemorrhagic lesion at early stage (Figure 5A). Interestingly, TER-119(+) regions were also accompanied by increased glial fibrillary acidic protein (GFAP) immunoreactivity (Figure 5B), which may be due to early astrogliosis responding to ICH (Perry et al., 2019). These results suggest two types of ICH lesions, SWI(+)/Gd(−) and SWI(+)Gd(−), are implicated in the development of Gould syndrome, though their chronological relationship warrants validation in larger studies.

## Discussion

Gould syndrome is a highly variable multisystem disorder caused by *COL4A1* and *COL4A2* mutations, whose clinical presentation can vary widely between and within families ranging from asymptomatic *COL4A1/COL4A2* mutation carriers to severely disabled individuals (Guey and Hervé, 2022). The key manifestation of Gould syndrome in adults is a wide range of cSVD conditions, including porencephaly, ICH of variable severity, and white matter lesions (Guey and Hervé, 2022; Vahedi, 2007; de Vries, 2009). Previously, we successfully demonstrated that *Col4a1* and *Col4a2* mutant mice can recapitulate pathophysiological hallmarks of Gould syndrome, including ICH, whose severity was modulated by allelic heterogeneity (Jeanne et al., 2015; Jeanne and Gould, 2017). In this study, we show that our multimodal MRI approach can detect radiological features of cSVD in *Col4a1* mutant mice that are similar to those observed in individuals with Gould syndrome, and extend our previous findings of allelic heterogeneity contributing to the variability of cerebrovascular manifestations in *Col4a1* mutant mice (Jeanne et al., 2015).

*COL4A1* mutations were initially discovered in humans as a genetic cause of porencephaly (Gould et al., 2005) and cSVD (Gould et al., 2006). Subsequent studies identified similar consequences for *COL4A2* mutations (Yoneda et al., 2012; Jeanne et al., 2012; Verbeek et al., 2012; Gunda et al., 2014). Large scale studies validated *COL4A1* mutations as frequent causes of porencephaly and schizencephaly and *COL4A1* and *COL4A2* as frequent causes of prenatal hemorrhagic or ischemic cerebral lesions (Maurice et al., 2021; Coste et al., 2022). Individuals with pathogenic variants in *COL4A1* and *COL4A2* had clinical stroke for which hemorrhagic events were the most common causes. Radiological findings include white matter hyperintensities, cerebral hemorrhage, ischemic lesions, and cerebral microbleeds. Less frequently reported findings included brain atrophy, enlarged perivascular spaces, calcification, and cerebral aneurysms (Whittaker et al., 2022). Missense mutations of highly conserved glycine residues in COL4A1 or COL4A2 triple helical domains are the most common class of mutation. Mice with *Col4a1* or *Col4a2* mutations model the human disease and demonstrate that genetic background and allelic heterogeneity both contribute to the variable expressivity (Jeanne et al., 2015; Labelle-Dumais, 2019; Kuo et al., 2014; Gould et al., 2007; Mao, 2015; Mao, 2021). Here we used different strains from a *Col4a1* allelic series in an attempt to model the breadth of clinical manifestations reported in human patients.

The typical neuroimaging lesions detected by our multimodal MRI approach included both enlarged ventricle size and ICH. Previous studies indicate that the enlarged ventricle might be secondary to the recurrence of ICH^47^. In line with this notion, dilated ventricles were mainly found among *Col4a1^+/G1038S^* mice, which also exhibited the highest ICH burden. This is consistent with previously reported histological findings using a larger murine allelic series of Col4a1 and Col4a2 mutations (Jeanne et al., 2015; Kuo et al., 2014). Meanwhile, the majority of ICH lesions across all mutant strains were located in periventricular subcortical regions, similar to what had been reported in individuals with Gould syndrome (Guey et al., 2021). This study also demonstrated that the *Col4a1* murine allelic series can model a range of ICH lesion characteristics, including prevalence, number, volume, and bleeding activity. In particular, by the age of 12-month old, *Col4a1^+/G1038S^* mice presented the highest lesion burden, the largest solitary lesion size, and the most extensive affected region among all *Col4a1* mutations examined in this study; however, only 1.3% of *Col4a1^+/G1038S^* showed Gd(+) which radiology feature indicates active ICH.

This multimodal imaging modality also detected porencephaly and schizencephaly, two radiological findings associated with Gould syndrome (Meuwissen, 2015), in Col4a1 mutant mice. Porencephaly was detected in one mouse in the *Col4a1^+/G1038S^* background but not in other genotypes (Supplementary Figure S3). To better characterize the lesion, the infiltration of CSF into the corpus callosum was confirmed through combined analysis of TW2I and FLAIR within the same brain slice. Considering porencephaly can cause severe consequences leading to early lethality, its higher prevalence may thus have been precluded in this study, which suggests future MRI study should be conducted at an earlier time point (<12 month) for the pathogenesis of porencephaly. Additionally, schizencephaly, although rarely reported in individuals with Gould syndrome (Meuwissen, 2015), was also detected with low incidence in Col4a1 mutant mice (one *Col4a1^+/G1038S^* mouse, Figure 3). Those two cases suggest that *Col4a1^+/G1038S^* mice may serve as an ideal pre-clinical platform to study the evolution of various radiological phenotypes of Gould syndrome, although a larger sample size is needed for a more comprehensive phenotypic description of other *COL4A1* and *COLA2* mutations. Notably, periventricular leukoencephalopathy, a frequent MRI finding in individuals with *COL4A1* and *COL4A2* mutations characterized by FLAIR hyperintensity, was not identified in this study. Since proton tends to have longer $T_{1}$ and $T_{2}$ values at ultra-high field and the signal-to-noise ratio (SNR) of inversion recovery sequence follows the equation $\mathrm{SNR}\propto (1-2e^{-T1/T_{1}}).(1-2e^{-\mathrm{TR}/T_{1}}).2e^{-\mathrm{TE}/T_{2}},$ the absence of white matter hyperintensity in this study could be attributed to the current FLAIR sequence lacking enough SNR to afford adequate tissue contrast at 14.1 T. Alternatively, leukoencephalopathy, like porencephaly or schizencephaly, may have a low prevalence in the *Col4a1* mutant mice studied here, and further large-scale studies with a wider age range and a more white matter-specific pathological validation (like Luxol Fast Blue staining) are needed to investigate this possibility.

This study was designed as a methodological exploration to establish an imaging platform to characterize phenotypic heterogeneity in a monogenic murine disease model. Now that the neuroimaging and analysis pipelines are established, future studies can characterize disease progression longitudinally for each genotype. In particular, longitudinal MRI can track the formation and evolution of ICH, making it highly efficient to study acute/subacute and chronic-stages of ICH by leveraging SWI and Gd-enhanced T1WI techniques, and to explore potential difference between genotypes.

In this study, two convolutional networks with U-net architecture were implemented to perform the brain structure segmentation on the acquired MRI data, enabling quantitative morphometric analysis. To prevent potential overfitting, we trained and validated both networks on a previously published mouse brain MRI dataset before applying the optimized model to the animals in this study. The first segmentation task was whole brain extraction, commonly referred to as skull-stripping, for which numerous automatic algorithms have been proposed for human brain imaging (Kalavathi and Prasath, 2016) but remained underdeveloped for preclinical research. Given that U-Net-Based segmentation has demonstrated robustness against inter-subject variability and efficiency in handling rodent brain MRI data (Hsu, 2020), it was chosen as an optimal architecture for MRI contrast-indifferent skull-stripping while limited dataset was available in this study. The loss function used in our convolutional networks is Local Weighting-Modified Dice coefficient, which was reported to offer greater robustness than the conventional Dice coefficient as for class-imbalanced segmentation tasks (Sugino, 2021). The same neural network architecture and loss function were used for ventricle segmentation, where both T2WI and FLAIR were fed as input data, which proved sufficient to differentiate CSF from brain tissue. This multi-modality input strategy was also applied in another U-net (not reported here) aimed at quantifying leukoencephalopathy lesions, however, the scarcity of such pathological changes in this study precluded sufficient training.

Although the multimodal MR imaging in this study was performed at non-clinical field strength, our findings suggest that multimodal MRI combined with machine learning holds significant promise for aiding individuals with Gould syndrome in a clinical setting. As demonstrated here, multimodal MRI enabled the identification of subtle brain changes, that potentially enable early detection of the disorder, and personalized treatment strategies tailored to each patient’s unique neurological profile. Moreover, machine learning algorithms can analyze vast amounts of multimodal MRI data to uncover complex patterns and biomarkers indicative of disease progression, facilitating timely interventions and potentially improving patient outcomes. By integrating multimodal MRI and machine learning, clinicians could enhance their understanding of Gould syndrome and develop more effective therapeutic interventions to alleviate its symptoms and improve the quality of life for affected individuals.

## Materials and methods

### Animals

All animal research was approved by the Institutional Animal Care and Use Committee of the University of California, San Francisco (protocols AN159737 and AN182181). We studied five strains of mice from a *Col4a1* allelic series (Kuo et al., 2014) (Figure 1A). The mutations affect four distinct glycine residues in the COL4A1 triple helical domain: G658D, G912V, G1038S, G1180D, and one missense mutation in a serine residue in the NC1 domain: S1582P. Each genotype group contained 5 male animals of 52.1 ± 1.26 weeks of age (Supplementary Figure S1A) and age- and sex- matched wild-type (WT) littermates (*n* = 5) were used as controls.

### MR acquisitions

The workflow of this multimodal MRI study is illustrated in Figure 1B. All *in vivo* MR experiments were conducted on a 14.1 Tesla vertical MR system (Agilent Technologies, Palo Alto, CA) equipped with 100G/cm gradients and a single tuned millipede ^1^H proton coil (inner diameter = 40 mm). For each imaging session, mice were anesthetized using isoflurane (1–1.5% in O_2_) and positioned in a dedicated cradle maintaining constant anesthesia and placed in the MR bore; respiration and temperature were continuously monitored during all acquisitions to ensure animal well-being and data reproducibility. Four optimized sequences of matching geometry (axial orientation, field of view (FOV) = 20 × 20 mm^2^, matrix = 256 × 256, 16 slices, 0.4 mm slice thickness, 0.1 mm interslice gap) were used during the same session for an overall scan time of ~60 min/animal. The sequences are briefly described below, while detailed parameters can be found in Table 2 (Wardlaw et al., 2013; Miura et al., 2017; Thaler et al., 2019; Wu et al., 2023).

1. Susceptibility Weighted Imaging (SWI) was used to detect potential ICH, as pathological lesions in Gould syndrome result in iron deposits (Gould et al., 2005; Gould et al., 2006), leading to main magnetic field (B_0_) inhomogeneity detectable by SWI.
2. T2-weighted imaging (T2WI) was performed using a fast-spin-echo (FSE) scheme, as FSE-based T2WI is less sensitive to distortion artifacts caused by B_0_ inhomogeneity than SWI, and is thus used for the atlas-based brain structure registration as described.
3. Fluid Attenuated Inversion Recovery (FLAIR) was used to quantify ventricle volumes and detect potential white matter hyperintensity lesions.
4. T1-weighted imaging (T1WI) w/o Gadolinium (Gd) injection was performed to detect potential defects in blood brain barrier integrity. The same GE scheme as SWI was used, but repetition time (TR) and echo time (TE) were shortened to increase sensitivity to T1 effect (linked to local concentration of Gd) and decrease sensitivity to T2* effect (due to local B_0_ inhomogeneity). Images were acquired before (pre-) and after (post-) injection of Gd through the tail vein (Gadavist®, 40𝜇L/100 g body weight, 1:4 diluted with normal saline).

**Table 2:** Description of MRI modalities.

MRI modality	Sequence parameters	Scan time	Readouts	Refs
T2-Weighted Imaging (T2WI)	Readout type: SE TE/TR = 21.38 ms/2500 ms FA = 90°, NEX = 8	5min12s	Anatomical structure Volume and intensity of brain regions High grey matter/white matter contrast	56–58
Susceptibility-Weighted Imaging (SWI)	Readout type: GE TE/TR: 21.38 ms/2500 ms FA = 10°, NEX = 16	9min35s	Highly sensitive to iron content Microbleeds/microhemorrhages	56–58
Fluid-Attenuated Inversion Recovery Imaging (FLAIR)	Readout type: Fast SE TE/TR = 14.26 ms/5500 ms, TI = 1,600 ms FA = 90°, NEX = 4	12min50s	White matter hyperintensities Differentiation between CSF and T2W hyperintense lesions	56–59
Gd-Enhanced T1-Weighted Imaging (Gd-Enhanced T1WI)	Readout type: GE TE/TR = 2.41 ms/120 ms FA = 40°, NEX = 10, Acquired pre/post Gd injection	4m7s	Blood–brain barrier disruption Active microhemorrhages	56,58

All four (T2WI, FLAIR, SWI and Gd-Enhanced T1WI) MR sequences share the same geometry: field of view (FOV) = 20 × 20 mm2, matrix size = 256 × 256, slice thickness = 0.4 mm, slice gap = 0.1 mm, number of slices = 16. Additional parameters optimized for 14.1 T imaging are described in the table. CSF, cerebral spinal fluid; FA, flip angle; Gd, Gadolinium; GE, gradient echo; NEX, number of excitations; SE, spin echo; TE, echo time; TI, inversion time; and TR, repetition time.

### Volumetric analysis and lesion quantification

For volumetric analysis, a U-net Convolutional Neural Network architecture (Ronneberger et al., 2015) was optimized and implemented in MATLAB®. Two separate U-Net architectures were included in this study: one was trained to generate a brain mask (‘skull-stripping’) by using any of the three MRI sequences (T2WI, T1WI, and SWI) as input. The other neural network was trained for segmentation of ventricle volume by using stacked T2WI-FLAIR data as input. The training dataset came from published data from our group that used the same MRI modalities on a different Gould syndrome mouse model (Yamasaki et al., 2023). The ground truth for segmentation training was manually labeled in ImageJ. The training parameters for both U-net architectures are listed in Supplementary Table S2. All segmentation outputs underwent a quality-control (QC) process by a human reader, where the off-target results were replaced by manual segmentation. Brain and ventricle volumes were extracted from U-net, adjusted *via* QC, and calculated for each animal. Ventricle-to-brain ratio was also calculated.

SWI-positive lesions were defined as hypointense pixels in SWI images and quantified using manual segmentation in ImageJ. The prevalence, number, and volume of lesions were compiled for each animal and for each group. To investigate the distribution of SWI-positive lesions across the brain, an open-sourced atlas-based imaging data analysis pipeline (AIDAmri) (Pallast et al., 2019) was customized to register T2W brain images to the Allen Brain Reference Atlas (Wang et al., 2020). Based on the registration result, the SWI-positive lesion volumes for each brain region were summed up across all animals and an intra-group region-wise volumetric analysis was performed using Excel (Microsoft, WA, United States).

### Histological and molecular characterization of SWI-positive lesions

Right after the MRI scan, all mice were maintained under anesthetized state and transcardially perfused with phosphate buffered saline (PBS) prior to organ collection. Brains were fixed by immersion in 4% paraformaldehyde overnight at 4 °C and cryoprotected in 30% sucrose in PBS at 4 °C for 48 h, embedded in optimal cutting tissue compound (Sakura Finetek, CA, USA) and flash frozen using dry ice. To validate the pathological nature of SWI positive lesions, 40 μm coronal cryosections parallel to the MRI slices were collected along the rostro-caudal axis and stained with Perl’s Prussian Blue and counterstained with nuclear Fast red (Vector Lab, CA, United States) as described previously (Jeanne et al., 2015; Hayashi et al., 2018). Images were acquired using a SteREO Discovery V8 microscope, AxioCam ICc3 camera and AxioVision 4.6 software (Zeiss, NY, United States).

Another set of 40 μm cryosections underwent incubation overnight at 4 °C with rat anti-TER-119 (1:500, R&D Systems, MN, United States) and rabbit polyclonal anti-GFAP antibody (1:1000, Dako, Glostrup, Denmark). The primary antibodies were detected using corresponding secondary antibodies after 2 h of incubation at room temperature. ProLong Gold containing 4′,6-diamidino-2-phenylindole (DAPI; Invitrogen, CA, United States) was used for mounting the sections. Images were acquired using the same microscopy system as described above.

### Statistical analysis

Statistical analysis was performed using a one-way ANOVA with Dunnett’s *post-hoc* test in Prism (GraphPad, MA, United States), comparing WT to each of the mutant genotype groups, for each of the following parameters: body weight, ventricle volume, brain volume, ventricle-to-brain ratio (*p < 0.05, **p < 0.01).

## Data Availability

The raw data supporting the conclusions of this article will be made available by the authors, without undue reservation.
